# Immunoexpression of the COX-2, p53, and caspase-3 proteins in colorectal adenoma and non-neoplastic mucosa

**DOI:** 10.1590/S1679-45082013000400009

**Published:** 2013

**Authors:** Renan Brito Nogueira, Andréa Rodrigues Cordovil Pires, Thélia Maria Santos Soares, Simone Rabello de Souza Rodrigues, Mariane Antonieta Menino Campos, Giovanna Canato Toloi, Jaques Waisberg

**Affiliations:** 1 Graduate Program in Gastrointestinal Surgery, Escola Paulista de Medicina, Universidade Federal de São Paulo, São Paulo, SP, Brazil; 2 Universidade Federal Fluminense, Niterói, RJ, Brazil; 3 Hospital Irmandade São João Batista, Macaé, Rio de Janeiro, RJ, Brazil; 4 Fonte Medicina Diagnóstica, Niterói, RJ, Brazil; 5 Faculdade de Medicina do ABC, Santo André, SP, Brazil

**Keywords:** Adenoma, Immunohistochemistry, Gene, p53, Caspase 3, Cyclooxygenase 2, Intestine, large

## Abstract

**Objective:**

To analyze the immunoexpression of the COX-2, p53, and caspase-3 proteins in colorectal adenomas and non-neoplastic mucosa.

**Methods:**

72 individuals were subjected to colonoscopy, which provided 50 samples of adenomas and 45 samples of non-neoplastic colorectal mucosa. The tissue samples were obtained via the tissue microarray technique and subjected to immunohistochemical analysis using primary anti-p53, anti-COX-2, and anti-caspase-3 antibodies. The positivity and intensity of the immunoreaction were classified. The analyzed variables were as follows: site of the adenomas in the colon, degree of dysplasia, size, and score of positivity and intensity of immunoexpression of the p-53, caspase-3, and COX-2 proteins.

**Results:**

The immunoexpression of mutated protein p53 was positive in 30 (60%) adenoma samples and negative in 20 (40%) adenoma samples. The immunoexpression of mutated protein p53 was negative in 39 (86.6%) samples and positive in 6 (13.3%) samples of the non-neoplastic colorectal mucosa (p<0.0001). Significant differences were seen between both the largest size (p=0.006) and the highest degree of dysplasia (p<0.0001) of the adenomas and the intensity of immunoexpression of mutated protein p53. The positivity and intensity of immunoexpression of COX-2 (p=0.14) and caspase-3 (p=0.23) showed no significant differences between the adenomas and the non-neoplastic colorectal mucosa.

**Conclusion:**

Mutated protein p53 was hyperexpressed in the adenomas compared with the non-neoplastic mucosa. Greater size and greater degree of dysplasia in the adenomas were associated with higher expression of mutated protein p53. The immunoexpression of COX-2 and caspase-3 in the adenomas did not exhibit a correlation with the anatomical-pathological features of the tumors and did not differ from the corresponding expression levels in the non-neoplastic mucosa.

## RESUMO

**Objetivo:**

Analisar a imunoexpressão das proteínas COX-2, p53 e caspase-3 em adenomas colorretais e na mucosa não neoplásica.

**Métodos:**

Foram submetidos à colonoscopia 72 indivíduos que forneceram 50 amostras de adenomas e 45 de mucosa colorretal não neoplásica. Os tecidos foram obtidos pela técnica de arranjo em matriz (*tissue microarray*) e submetidos a estudo imunoistoquímico com anticorpos primários p53, COX-2 e caspase-3. A positividade e intensidade da imunorreação foram classificadas. Foram estudadas as seguintes variáveis: localização do adenoma no colo, grau de displasia, tamanho, e escores de positividade e intensidade da imunoexpressão das proteínas p-53, caspase-3 e COX-2.

**Resultados:**

Nos adenomas, a imunoexpressão da proteína p53 mutada foi positiva em 30 (60%) e negativa em 20 (40%) amostras. Na mucosa colorretal não neoplásica, a imunoexpressão da proteína p53 mutada foi negativa em 39 (86,6%) amostras e positiva em 6 (13,3%) (p<0,0001). Houve diferença significativa entre o maior tamanho (p=0,006) e o maior grau de displasia dos adenomas (p<0,0001) e a intensidade de imunoexpressão da proteína p53 mutada. A positividade e intensidade da imunoexpressão das proteínas COX-2 (p=0,14) e caspase-3 (p=0,23), nos adenomas e na mucosa colorretal não neoplásica, não apresentaram diferença significante.

**Conclusão:**

A proteína p53 mutada é hiperexpressada nos adenomas em comparação com a mucosa não neoplásica. Nos adenomas, o maior tamanho e o maior grau de displasia foram associados à maior expressão da proteína p53 mutada. A imunoexpressão das proteínas COX-2 e caspase nos adenomas não apresentou correlação com os aspectos anatomopatológicos e não foi diferente em termos de níveis de expressão correspondentes na mucosa não neoplásica.

## INTRODUCTION

Genes upregulated in adenoma relative to normal tissue, which maintained increased expression in colorectal carcinoma and adenoma, would encode proteins suitable as putative targets for immunoprevention^(1,2)^. The identification of early and easily detectable tumor markers, that might contribute to the treatment of colorectal carcinogenesis, as the biological mechanisms required for preinvasive adenoma to progress to carcinoma, is highly relevant subject^(3,4)^. Despite constant improvement of staging methods for colorectal carcinoma, we also observed a high degree of unpredictability of results, demonstrating a greater need for knowledge of determinants of the evolution of neoplasia^(5–8)^.

Gene p53, which is considered a DNA replication inhibitor, participates in regulating apoptosis and blocking angiogenesis^(2,9)^ and is considered a tumor suppressor gene. Mutations in this gene occur frequently in human cancer with important implications for cell apoptosis^(10)^. Lung, breast, and colon cancers are frequently associated with mutations in p53 and hyperexpression of the encoded protein^(9,10)^.

Caspase-3 plays an important role in intrinsic (or mitochondrial) and extrinsic (or cytoplasmic) activation pathways of apoptosis. The protein's expression in the non-neoplastic mucosa and colorectal adenomas is a controversial issue in the literature^(6,11,12)^.

Cyclooxygenase-2 (COX-2) participates in the response to inflammatory stimuli, growth factors, angiogenesis, hormones, mitogenesis, and carcinogenesis^(9–16)^. COX-2 inhibitors might reduce tumoral angiogenesis and promote apoptosis^(17,18)^. Studies with animal and human models demonstrated that the use of COX-2 inhibitors can prevent or hinder the adenoma-carcinoma progression^(17,19)^. The expression of COX-2 is significantly increased in tumor tissues^(9,13)^. However, the mechanism behind the protective effect of COX-2 inhibitors against the normal mucosa-adenoma-carcinoma sequence has not been fully elucidated to date^(17,19)^.

## OBJECTIVE

To analyze the immunohistochemical expression of the p53, COX-2, and caspase-3 proteins in colon adenomas and in the non-neoplastic colorectal mucosa and to determine the possible association of this expression with the anatomical-pathological features of the neoplasm.

## METHODS

### Study design

The study was approved by the Research Ethics Committee (number 0279/10) of *Escola Paulista de Medicina da Universidade Federal de São Paulo* (UNIFESP).

The present investigation was a retrospective study (from January 2005 to December 2006) that included biopsy samples of colorectal adenomas and non-neoplastic mucosa resected during colonoscopies and removed from paraffin blocks.

### Sample characteristics

Included were adult patients of both genders with colorectal adenomas that were diagnosed upon anatomical-pathological examination. The exclusion criteria were patients with colorectal adenomas associated to colorectal carcinomas, inflammatory bowel diseases, colorectal polyposis syndrome, and age <18 years.

Ninety-five paraffin-block samples from 72 patients subjected to colonoscopy were obtained from THELAB Pathology Laboratory (Macaé, RJ, Brazil) and were analyzed. The samples were allocated to two groups: Group adenoma (GA), which comprised 40 patients with colorectal adenoma, and Group Control (GC), which comprised 32 patients without colorectal adenoma. Fifty adenoma samples were harvested from GA, and 45 samples of non-neoplastic mucosa were harvested from GC.

GA included 21 (52.5%) males and 19 (47.8%) females, with a mean age of 57.8±13.1 years (28 to 83 years). GC included 17 (53.1%) males and 15 (46.9%) females with a mean age of 46.1±14.9 years (18 to 74 years).

### Tissue samples and immunohistochemistry

Slides of the paraffin blocks were prepared and stained with hematoxylin-eosin (HE). The selected blocks were subjected to the tissue microarray (TMA) technique.

The TMAs were prepared according to the technique described by Pires et al.^(20,21)^. The TMA blocks and immunohistochemical analyses were performed at the Fonte Pathology Laboratory (Niterói, RJ, Brazil).

To ensure the representativeness of each area selected in the paraffin blocks for immunohistochemical analysis, two samples were collected from different sections of the same block.

To prepare the histological slides, the TMA paraffin blocks were cut into 3-*μ*m-thick sections and stained with HE. The immunohistochemical technique was performed according to protocols previously established by the laboratory. The following primary antibodies were used on the TMA slides: anti-protein p53 (clone SP5, code 453R-14, Cell Marque Corp., Rocklin, California, USA), caspase-3 (polyclonal, code CP229A, Biocare Medical, Concord, California, USA), and COX-2 (clone SP21, code 240R-14, Cell Marque Corp., Rocklin, California, USA). External cases (placenta, kidney, and liver) were added to each slide to identify the position of each case in the TMA.

For the immunohistochemical reactions, 3-*μ*m-thick histological sections were obtained and placed on histological slides previously treated with saline solution (Sigma Chemical Co., St. Louis, Missouri, USA), according to protocols previously established by the laboratory.

The intensity of the expression of p53, COX-2, and caspase-3 on the slides was semi-quantified as follows: negative or zero, mild or 1 (<10% stained cells), moderate or 2 (10 to 50% stained cells), and strong or 3 (>50% stained cells)^(22)^. The microscopic analysis of the slides was performed under an optical microscope (Eclipse E200, Nikon, Japan), with a 400X final magnification. The HE-stained slides and the TMA slides were examined by two pathologists. The positive controls used for immunohistochemical analysis were tissues from human tonsils. As a negative control, the primary antibody was removed from the immunohistochemical reaction.

### Data collection

The results were analyzed based on the following variables: size, site, histological type, and degree of dysplasia of the adenomas, as well as the intensity and distribution of the immunoexpression of p53, caspase-3, and COX-2 in the colorectal adenomas and non-neoplastic mucosa.

### Statistical analysis

Descriptive statistics were summarized as mean ± standard deviation (SD) or frequencies (percentages), as appropriate. The χ^2^ test was used for qualitative variables, *e.g.*, frequencies and proportions. The statistical software used was Statistical Package for the Social Sciences (SPSS) for Windows, version 10.1 (SPSS Inc., USA). The level of significance was established as 5%.

## RESULTS

The mean size of the adenomas (GA) was 0.62±0.2mm (2 to 20mm). Of the 50 adenomas, 29 (58%) were tubular, 17 (34%) tubulovillous, and 4 (8%) villous. Dysplasia was mild in 25 (50%) tumors, moderate in 18 (36%), and severe in 7 (14%). The most frequent site of the adenomas was the sigmoid colon, accounting for 17 (34%) tumors, followed by the transverse colon with 11 (22%), descending colon with 7 (14%), cecum with 7 (14%), ascending colon with 4 (8%), and rectum with 4 (8%).

Comparison of the immunoexpression of the p53, COX-2, and caspase-3 proteins between the colorectal adenomas and the non-neoplastic mucosa yielded a significant difference (p<0.0001) in the immunoexpression of p53 between GA and GC, whereas the expression of COX-2 (p=0.14) and caspase-3 (p=0.24) did not exhibit a significant difference (Table 1).

**Table 1:** Immunoexpression intensity of the p53, COX-2, and caspase-3 proteins in colorectal adenomas and the non-neoplastic mucosa

Expression level	Proteins					
	p53		COX-2		Caspase-3	
	Groups n (%)		Groups n (%)		Groups n (%)	
	C	A	C	A	C	A
0	39 (88.6)	20 (40)	0 (0.0)	0 (0.0)	0 (0.0)	1 (2)
1	3 (6.8)	10 (20)	9 (20.0)	10 (20.4)	9 (20.0)	5 (10)
2	1 (2.3)	14 (28)	20 (44.4)	30 (61.2)	23 (51.1)	22 (44)
3	1 (2.3)	6 (12)	16 (35.6)	9 (18.4)	13 (28.9)	22 (44)
Total	44§	50	45	49§	45	50
p value	<0.0001*		0.14 (ns)		0.23 (ns)	

χ^2^ test.

§ One sample was lost;

* significant.

A: group with colorectal adenomas; C: group with non-neoplastic mucosa; ns: non-significant.

Significant differences were found (p<0.0001) between the immunoexpression of p53 in the various histological types of colorectal adenoma and the corresponding immunoexpression in the normal mucosa (Table 2).

**Table 2:** Number of adenomas and the corresponding percentage of the p53 expression level in patients with non-neoplastic colorectal mucosa (control) and patients with tubular, tubulovillous, and villous adenomas

Expression level	Protein p53 n (%)				Total n
	Control	TA	TVA	VA	
0	39 (88.6)	14 (48.3)	6 (35.3)	0 (0)	59
1	3 (6.8)	5 (17.2)	5 (29.4)	0 (0)	13
2	1 (2.3)	8 (27.6)	5 (29.4)	1 (25)	15
3	1 (2.3)	2 (6.9)	1 (5.9)	3 (75)	7
Total	44§	29	17	4	94

χ^2^ test; p<0.0001.

§ One sample was lost.

TA: tubular adenomas; TVA: tubulovillous adenomas; VA: villous adenomas; VA: villous adenoma.

No significant association was found between the immunoexpression of COX-2 (p=0.08) and caspase-3 (p=0.12) in t he various histological types of colorectal adenomas and normal mucosa (Tables 3 and 4).

**Table 3:** Number of samples and corresponding percentage of the COX-2 expression level in patients with non-neoplastic colorectal mucosa (control) and patients with tubular, tubulovillous, and villous adenomas

Expression level	Protein COX-2 n (%)				Total n
	Control	TA	TVA	VA	
0	0 (0.0)	0 (0.0)	0 (0.0)	0 (0.0)	0
1	9 (20.0)	5 (17.2)	3 (18.7)	2 (50.0)	19
2	20 (44.4)	18 (62.1)	11 (68.8)	1 (25.0)	50
3	16 (35.6)	6 (20.7)	2 (12.5)	1 (25.0)	25
Total	45	29	16	4	94

χ^2^ test; p=0.29. COX-2: cyclooxygenase-2; TA: tubular adenomas; TVA: tubulovillous adenomas; VA: villous adenomas.

**Table 4:** Number of samples and the corresponding percentage of the caspase-3 expression level in patients with non-neoplastic colorectal mucosa (control) and patients with tubular, tubulovillous, and villous adenomas

Expression level	Protein caspase-3 n (%)				Total n
	Control	TA	TVA	VA	
0	0 (0.0)	1 (3.5)	0 (0.0)	0 (0.0)	1
1	9 (20.0)	3 (10.3)	1 (5.8)	1 (25.0)	14
2	23 (51.1)	14 (48.3)	8 (47.1)	0 (0.0)	45
3	13 (28.9)	11 (37.9)	8 (47.1)	3 (75.0)	35
Total	45	29	17	4	95

χ^2^ test; p = 0.44. TA: tubular adenomas; TVA: tubulovillous adenomas; VA: villous adenomas.

The association between the intensity of p53 immunoexpression and the degree of dysplasia of the colorectal adenomas was significant (p=0.03). The relationship between the intensity of immunoexpression of COX-2 and caspase-3 and the degree of dysplasia of the colorectal adenomas was not significant (p=0.76 and p=0.3, respectively).

The association between the distribution of the mean size of the colorectal adenomas and the immunoexpression of p53, COX-2, and caspase-3 showed a significant difference (p=0.006).

## DISCUSSION

Patients with adenomas exhibit a threefold increased risk of developing colorectal cancer, a risk that is even greater among patients who are older than 60 years and/or present with multiple colorectal adenomas^(1)^. However, the relation between the histogenesis of colorectal cancer and genetic alterations, as well as the accumulation of biomarkers, is not sufficiently understood to date^(2,3)^.

Most of the research on this subject has focused on the genes controlling apoptosis^(4,5)^, which is a target of cancer treatment at several stages of the progression of tumors. Disorders in the regulation of the cell cycle and pathways of apoptosis occur frequently in the colorectal mucosa-adenoma-carcinoma sequence^(5–8)^.

The present study assessed the expression of the p53, COX-2, and caspase-3 proteins in tubular, tubulovillous, and villous colorectal adenomas, as well as in the non-neoplastic mucosa of the colon, by means of immunohistochemical analysis using the TMA technique.

Immunohistochemical expression of p53 occurs when it is inactive, and the protein accumulates inside the cell nuclei, which might be caused by mutations in the p53 gene or by protein inactivation mediated by other molecules, which by hindering the transformation of p53 from its monomeric to its tetrameric form, prevent p53 from binding to DNA and impede functionality of this protein^(23)^.

The presence of a mutated p53 gene is frequent in the areas with greatest dysplasia of the adenomas^(23)^. Kaklamanis et al.^(23)^ assessed the expression of p53 in 72 adenomas as to their size, histological type, and degree of dysplasia and demonstrated that only the latter exhibits a significant association with the expression of p53. The immunoexpression of p53 is absent in the non-neoplastic colorectal mucosa. In this study, the p53 protein was not expressed in 39 (88.63%) of the non-neoplastic mucosa samples. Regarding the size of the adenomas, greater expression of p53 was found in the largest tumors, confirming the results of Kaklamanis et al.^(23)^.

Sheikh et al.^(1)^ analyzed the expression of p53 in 42 adenomas with high degrees of dysplasia, 15 of which also exhibited carcinomas, and found the expression of p53 in 27 (64.2%) cases. Among the adenomas with *in situ* carcinomas, 93% were p53-positive, whereas 48% of the colorectal adenomas without *in situ* carcinoma were p53-positive. Ieda et al.^(24)^ studied the expression of p53 in 139 colorectal adenomas, 57 colorectal adenomas with early carcinoma, and 12 samples of colorectal carcinoma. The results revealed a significantly higher level of p53 expression in the adenomas with a greater degree of dysplasia. Visca et al.^(25)^ analyzed the immunoexpression of apoptosis-regulatory proteins (including p53) in one hundred adenoma samples, one hundred carcinoma samples, and one hundred samples of adjacent non-neoplastic mucosa. The p53 protein was not expressed in any samples from the non-neoplastic mucosa. The adenomas with a high degree of dysplasia exhibited higher expression levels of p53. Sheikh et al.^(1)^ and Ieda et al.^(24)^ showed that the p53 protein expression increases with a higher degree of adenoma dysplasia. But these authors did not compare the results in tumors with non-neoplastic tissue. Visca et al.^(25)^ compared the expression of p53 protein in adenomas, carcinomas, and non-neoplastic mucosa adjacent to colorectal carcinoma. These authors studied formalin-fixed, paraffin-embedded archival material from one hundred non-consecutive adenomas and one hundred adenocarcinomas, including adjacent-to-tumor non-neoplastic mucosa, and negative controls were obtained from colon resections for non-neoplastic disease. They suggested that the evaluation in concert of clinicopathological data and immunohistochemical markers on both normal and abnormal colon tissue provides an attractive model of tumor progression. In the study, we observed that p53 protein expression was higher in adenomas than in the colorectal mucosa of patients without adenomas or carcinomas, and the adenoma size correlated with increased expression of p53 protein.

Leonardos et al.^(26)^ observed differences in the activity of caspase-3 in colorectal carcinomas compared with the non-neoplastic mucosa. The activity of caspase-3 was significantly higher in the tumor tissue compared with the non-neoplastic mucosa. Guan et al.^(12)^ found greater expression of caspase-3 in adenomas compared with the non-neoplastic colonic mucosa. However, Sena et al.^(11)^ studied the expression of caspase-3 in microadenomas and the non-neoplastic colonic mucosa and determined low levels of active protein in the microadenomas. Inappropriate or reduced functioning of the apoptotic process might represent an important characteristic of tumor progression. In the present case series, the expression of caspase-3 did not significantly differ between the adenomas and the non-neoplastic mucosa or as a function of the anatomical-pathological features of the adenomas.

Sato et al.^(14)^ conducted a retrospective study assessing the expression of COX-2 in 95 adenomas and the adjacent colorectal mucosa and found greater expression of COX-2 in the adenomas that exhibited higher degrees of dysplasia (with increased cell proliferation). McLean et al.^(13)^ assessed the expression of COX-2 in adenomas and the colorectal mucosa, and did not find expression of COX-2 in the latter, whereas the expression was significantly higher in the adenomas compared with the normal mucosa. In that study, the COX-2 expression level was higher in adenomas >10mm and in adenomas with greater degrees of dysplasia. Sheehan et al.^(9)^ assessed the expression of COX-2 in 123 adenomas and its relation with malignant transformation and found increased COX-2 expression levels that were proportional to the size, histological type, and degree of dysplasia of the adenomas. Wasilewicz et al.^(19)^ evaluated the expression of COX-2 in colon polyps and found higher expression levels in the adenomas than in the non-adenomatous polyps. Greater expression of COX-2 was found in the highly dysplastic adenomas and in polyps that were larger than 6mm. However, Einspahr et al.^(27)^ observed no significant relationship between the expression of COX-2 and the degree of dysplasia, the size, or the histological type upon analyzing 108 colorectal adenomas. Nevertheless, when the histological type and size (>7mm) of the adenomas were analyzed jointly, the expression levels of COX-2 exhibited a significant increase.

Sakuma et al.^(28)^ analyzed the expression of COX-2 in 21 patients with colorectal cancer. COX-2 was expressed in 8 (38.1%) samples, and there was no relation between the distribution and intensity of expression of this marker. Nakajima et al.^(29)^ found increased expression levels of COX-2 in tissue samples of carcinoma and in the non-neoplastic colonic mucosa. In the present study, expression of COX-2 was found in the non-neoplastic mucosa and the colorectal adenomas, although without a significant difference.

Han et al.^(30)^ correlated the expression of COX-2 with the tumor size in 50 colorectal adenomas and 40 carcinomas and did not determine a significant relationship. In the present study, the association between the expression of COX-2 and the degree of dysplasia, size, and histological type of the adenomas was not significant. This lack of association is possibly observed because the size of most of the adenomas was <10mm, and there were only a few villous adenomas in the sample.

The strategy for treating colorectal carcinoma by activating apoptosis in tumor cells specifically expressing the receptors for proteins related to cell death induction is attractive. Promising results are expected from ongoing phase I/II clinical trials that prove the efficacy of this therapy with agonist antibodies and/or recombinant proteins, alone or in combination with chemotherapeutic drugs^(31)^. However, fundamental questions need extensive studies before clinical application of this therapeutic modality can be considered safe. Their action must be selective and effective to reduce excessive systemic toxicity compared to normal cells. Moreover, better understanding of the signaling mechanisms triggered by genes that lead to the survival of cells resistant to apoptosis is needed^(31)^.

## CONCLUSION

Mutated protein p53 was hyperexpressed in the adenomas compared with the non-neoplastic mucosa. Increased adenoma size and degree of dysplasia were associated with higher expression of mutated p53. The immunoexpression of cyclooxigenase-2 and caspase-3 in the adenomas did not exhibit association with the pathological features of the tumor, and did not differ from the expression of these proteins in the non-neoplastic colorectal mucosa.
